# Editorial: Unconventional myosins in motile and contractile functions: fifty years on the stage

**DOI:** 10.3389/fphys.2024.1439746

**Published:** 2024-06-12

**Authors:** Manuel H. Taft, Maria Jolanta Redowicz

**Affiliations:** ^1^ Institute for Biophysical Chemistry, Hannover Medical School, Hannover, Germany; ^2^ Laboratory of Molecular Basis of Cell Motility, Nencki Institute of Experimental Biology, Polish Academy of Sciences, Warsaw, Poland

**Keywords:** unconventional myosins, physiology, cytoskeleton, molecular motor, myosin-1, myosin-6, myosin-5, myosin-18

The myosin superfamily of molecular motors can be classified by various means, of which the most classic one is the overall discrimination between “conventional” myosins that power muscle contraction and “unconventional” myosins that encompass all other myosins (Sellers, 2000; Berg et al., 2001; Odronitz and Kollmar 2007). After the first description of a muscle protein extraction termed “myosin” in 1864 (Kühne, 1864), all characterized myosins for more than a century were conventional myosins. Myosins, still generally mainly known for their involvement in muscle contraction, are molecular motors converting energy from ATP hydrolysis into movement along actin filaments. The first report on the isolation of skeletal muscle myosin was published in 1939 and it took several decades to show the presence of myosins in other muscle types and in non-muscle systems (Engelhardt and Liubimova, 1939; Sellers, 2000). A breakthrough in myosin research came in 1973 when Pollard and Korn purified from a protist, *Acanthamoeba castellanii*, one-headed myosin that was able to hydrolyze ATP in an actin-dependent manner but in contrast to muscle myosins was not capable of filament formation (Pollard and Korn, 1973a; Pollard and Korn, 1973b). The authors termed this novel myosin “myosin I” as unlike two-headed muscle myosins (“myosin II”) it contained only one head (Pollard and Korn, Front Physiol 2023). This discovery, considered by the journal *Nature* as one of the milestones in cell biology (Le Bot, 2010), opened a new era in studies on myosins. Intensive research, supported by a concomitant development of genetic, molecular biology, biophysical and visualization techniques, allowed for the discovery of a panoply of new myosins, which, similarly to *Acanthamoeba* myosin I, are not able to form filaments and thus have been termed unconventional ones. Arguably, this classification initially appears rather artificial, as it is not clearly based on phylogenetic, structural or functional parameters; yet it provides researchers with an easy organization, which mainly recognizes the historic context.

In this Research Topic, dedicated to celebrate the 50th anniversary of discovery of the first unconventional myosin, we aim to shed light on their known and novel roles and functions as exemplified by research papers and reviews on seven members, namely, myosin I, myosin IIIA, myosin V, myosin VI, myosin VIIA, myosin XVA and myosin XVIII.

As already emphasized by the title of our collection, one focus is on the historical perspective of research on unconventional myosins. We therefore invited Tom Pollard and Ed Korn to contribute their personal view on the discovery of the very first unconventional myosin, *Acanthamoeba* myosin I. The authors gladly followed our invitation and provided their article enlightening the reader about the laborious experimental process of the search for and ultimate discovery of *Acanthamoeba* myosin I (Pollard and Korn, Front Physiol 2023). The initial skepticism of many researchers about the purified novel myosin being a degradation product of a conventional myosin, as well as the presence of a cofactor necessary to establish ATPase activity, later identified as myosin heavy chain kinase (Maruta and Korn, 1977), further illustrates the long way to acceptance of the presence of unconventional myosins. We are sad to say that Ed Korn passed shortly after publishing his last article but we are sure that he and his discoveries will be remembered, not only by the myosin community.

We asked Jim Spudich to provide us with his personal view on the history of the most widely used experimental tools to study the motor function of myosins, the *in vitro* motility assay and the dual-beam single-molecule laser trap assay. In his article, he not only meticulously describes the invention, improvement and refinement of these aforementioned methods, but also acknowledges a large number of researchers who contributed to the success (Spudich, Front Physiol 2024).

The undoubtedly most studied and best-characterized unconventional myosin is myosin V, implemented in intracellular transport processes including organelle trafficking, vesicle and receptor transport and dynamic tethering and positioning of macromolecules, organelles and subcellular structures. The review article by Rüdiger Rudolf summarizes the vast present literature on the role of myosin Va, one of the three mammalian isoforms, in receptor recycling at the nerve-muscle synapse and discusses a concept of myosin V mediated vesicle tethering in active cAMP microdomains (Rudolf, Front Physiol 2024).

The functional diversification of class 5 myosins can further be increased by alternative splicing. In their research article, Carew et al. report the presence and distribution of Myo5a splice variants, produced from six small alternative exons, in rodent pelvic organs (Carew et al., Front Physiol 2024).

Whereas mammalian myosin 5a and 5b have been shown to move processively along actin filaments to enable cargo transport, myosin 5c appears to lack this property and is characterized as a low duty ratio non-processive motor that needs to cluster on the surface of cargo to support its continuous movement. Kengyel et al. found human myosin 5c localizing in pancreatic β–cells and added new insights on the regulation of the purified protein by characterizing the fine-tuning of its enzymatic and motor function by the presence of different cytoplasmic tropomyosin isoforms (Kengyel et al., Front Physiol 2024). In addition, the interaction of calmodulin as well as essential and regulatory light chains with myosin 5c modulating motor function and the small-molecule mediated inhibition by the allosteric class-5 myosin inhibitor, Pentabromopseudilin, are described.

The duty ratio of a myosin, reflecting the relative proportion of strongly actin bound states during its ATPase cycle, is a critical determinant for its mechanical function. Diensthuber et al. (Diensthuber et al., Front Physiol 2024) utilized mutations of a single amino acid (Y/F) in switch-1 of the active site of two *Dictyostelium discoideum* class-1 myosins to show that this position fine-tunes their kinetic signatures to modulate the respective duty ratio.

Myosin VI is the only myosin characterized so far which moves backwards on actin filaments, i.e., towards the minus end of the filament. It is mainly known from its involvement in endocytosis and cytoskeleton organization but besides its cytoplasmic functions has been shown to function within the nucleus (Shahid-Fuente and Toseland, 2023). Herein, Nowak et al. demonstrate that myosin VI is also present within the nucleolus and is involved in the maintenance of nucleolar structure and ribosome organization (Nowak et al., Front Physiol 2024).

The majority of reports on myosin VI comes from rodent and human studies. Behbehani et al. present new data describing two *Caenorhabditis elegans* myosin VI isoforms, SPE-15/HUM-3 and HUM-8. The authors show that while they share similar motor properties, they have distinct developmental and tissue expression patterns (Behbehani et al., Front Physiol 2024).

Class-18 myosins are acknowledged as being the most divergent members of the myosin superfamily. For both mammalian isoforms, Myo18A and Myo18B, neither ATPase activity nor motor function could be shown (Guzik-Lendrum et al., 2013; Taft et al., 2013; Latham et al., 2020); yet, these myosins are found in active contractile structures including muscle sarcomeres and stress fibers. Horsthemke et al. contribute a review article on the current knowledge of myosin-18 function, specifically discussing the role of splice isoform Myo18Aγ and Myo18B in the sarcomere (Horsthemke et al., Front Physiol 2024).

Several unconventional myosins are known to be involved in the inner ear functions as numerous mutations in their genes were found to be associated with deafness. Miyoshi et al. present a review addressing the mechanisms of myosins’ trafficking in a stereocilium using their motor function and make correlations between each described myosin variant with a clinical condition including the severity and onset of hearing loss, mode of inheritance and presence of symptoms other than hearing loss (Miyoshi et al., Front Physiol 2024). The authors concentrate on genes encoding unconventional myosins: IIIA, VI, VIIA and XVA, as well as on those encoding two conventional myosins - so called non-muscle myosins IIA (NM2A) and IIC (NM2C).

In summary, our Research Topic highlights recent discoveries on unconventional myosins (five research articles) and presents reviews (five articles) not only on particular myosins but also provides a historic perspective of renowned researchers on the discovery of the first unconventional myosin and the development of methods enabling studies on a single myosin molecule.
